# Expression of HGF and c-Met Proteins in Human Keratoconus Corneas

**DOI:** 10.1155/2015/852986

**Published:** 2015-11-30

**Authors:** Jingjing You, Li Wen, Athena Roufas, Chris Hodge, Gerard Sutton, Michele C. Madigan

**Affiliations:** ^1^Save Sight Institute, Discipline of Clinical Ophthalmology, The University of Sydney, Sydney, NSW 2000, Australia; ^2^Vision Eye Institute, Chatswood, NSW 2067, Australia; ^3^Auckland University, Auckland 1010, New Zealand; ^4^School of Optometry & Vision Science, UNSW, Kensington, NSW 2052, Australia

## Abstract

Keratoconus (KC) is a progressive degenerative inflammatory-related disease of the human cornea leading to decreased visual function. The pathogenesis of KC remains to be understood. Recent genetic studies indicate that gene variants of an inflammation-related molecule, hepatocyte growth factor (*HGF*), are associated with an increased susceptibility for developing KC. However HGF protein expression in KC has not been explored. In this initial study, we investigated late-stage KC and control corneas for the expression of HGF and its receptor mesenchymal-epithelial transition factor (c-Met/Met). KC buttons (~8 mm diameter) (*n* = 10) and whole control corneas (*n* = 6) were fixed in 10% formalin or 2% paraformaldehyde, paraffin embedded and sectioned. Sections were immunolabelled with HGF and c-Met antibodies, visualised using immunofluorescence, and examined with scanning laser confocal microscopy. Semiquantitative grading was used to compare HGF and c-Met immunostaining in KC and control corneas. Overall, KC corneas showed increased HGF and c-Met immunostaining compared to controls. KC corneal epithelium displayed heterogeneous moderate-to-strong immunoreactivity for HGF and c-Met, particularly in the basal epithelium adjacent to the cone area. Taken together with the recent genetic studies, our results further support a possible role for HGF/c-Met in the pathogenesis of KC.

## 1. Introduction

Keratoconus (KC) is the most common primary human degenerative corneal disease with a prevalence of around 1 in 2000 worldwide [1]. It is bilateral, asymmetric, and progressive, leading to corneal thinning and irregularity [2]. Onset primarily occurs in the 2nd decade of life and is associated with significant decreasing visual function [2] and morbidity [3]. KC is the main indication recorded for corneal grafts in Australia [4], and currently its progression can only be halted through surgical interventions including collagen cross-linking that stiffens the cornea using riboflavin and UVA [5]. More recently a surgical procedure was developed transplanting isolated Bowman's layer from donor corneas to KC eyes as a further late-stage intervention [6].

The histopathology of KC is well described and includes epithelial and stromal thinning within the apical cone region, breaks in the Bowman's layer, focal fibrosis, and anterior stromal keratocyte apoptosis [2, 7]. However the underlying pathogenesis of KC remains unclear. Recent evidence indicates a role for inflammation in the disease, with increased recruitment of inflammatory cells (e.g., macrophages, lymphocytes, and antigen presenting cells) [8] and inflammatory markers such as interleukin-1 (IL-1) and transforming growth factor-beta (TGF-*β*) [9] observed in KC corneal tissue sections. Increased expression of inflammatory markers such as interleukin-6 (IL-6), tumour necrosis factor alpha (TNF-*α*), and matrix metalloproteinase 9 (MMP-9) has also been found in tears collected from KC patients compared to controls [10]. Furthermore, a recent review examining the biochemical changes in KC proposed a “two-hit hypothesis” with a “genetic predisposition to the corneal disease and a second hit that may induce abnormalities of inflammatory components” [11].

Single nucleotide polymorphism (SNP) refers to a change in a single nucleotide within a DNA sequence and is the most common type of genetic variation observed in the human genome [12]. SNPs have been widely studied as genetic markers for human disease. Two parallel genome-wide association studies identifying potential SNPs associated with KC, using independent sample cohorts, reported a significant association between KC and the hepatocyte growth factor (*HGF*) gene, identifying two single nucleotide polymorphisms (SNPs; rs3735520 and rs17501108) in the promoter region [13]. Further, Burdon et al. (2011) also examined HGF protein abundance in the serum of controls correlating to the rs3735520 genotype and found a significant increase in HGF serum protein associated with the minor allele T [13].

HGF is a pleiotropic growth factor that activates the HGF/c-Met pathway after binding to its receptor, mesenchymal-epithelial transition factor (c-Met/Met). Once activated, downstream pathways such as mitogen-activated protein kinase (MAPK) cascades, PI3K-Akt axis or Janus kinase/signal transducers, and activators of transcription (JAK/STAT) pathways may be activated [14]. HGF has been implicated in several cellular roles within the cornea. For example, together with MMP-1, HGF is reported to initiate human corneal epithelial cell migration* in vitro* [15], and exogenous HGF has been found to promote the proliferation of both corneal epithelial and endothelial cells [16]. In injured rabbit corneas, Wilson et al. (1999) reported an obvious increase of* HGF* mRNA expression in keratocytes and* c-Met* mRNA expression in epithelial cells compared to unwounded corneas, suggesting that the HGF/c-Met pathway plays a role in corneal wound healing [17]. Studying bovine corneal wound healing in organ culture models, Carrington and Boulton (2005) showed that HGF delayed epithelial layer formation, together with increased differentiation of keratocytes to myofibroblasts, compared with untreated and keratinocyte growth factor (KGF) treated corneas [18].

The expression of HGF and c-Met proteins in human KC corneas has not been investigated to date. One study has reported increased serum HGF expression for at least the minor allele of HGF SNP rs3735520, associated with increased potential for developing KC [13]. As a first step in assessing the role of HGF protein and its receptor (c-Met) in KC, we used corneal buttons from patients with severe KC and control human corneas to compare and examine the distribution and expression of these proteins.

## 2. Materials and Methods

KC corneal tissue buttons (Vision Eye Institute, Chatswood, NSW Australia) and donor corneas (Lions New South Wales (NSW) Eye Bank) were obtained with consent and approval from the Sydney Eye Hospital Human Research Ethics Committee (HREC 2013/1041). All procedures were in accordance with the Declaration of Helsinki. Informed consent was obtained from all participants prior to collection of KC buttons. Normal donor corneas were obtained from the Lions NSW Eye Bank following appropriate consent and HREC approval.

### 2.1. Corneal Specimens

Ten corneal buttons were collected from KC patients (age range from 18 to 32 years) undergoing corneal transplantation at Vision Eye Institute. All KC patients were diagnosed on the basis of clinical signs and corneal topography and were classified as KC grade 4 (most severe stage) (Table 1). Six normal donor corneas (age range 53 to 83 years) were obtained from the Lions NSW Eye Bank (Table 2).

KC corneal buttons (~8 mm diameter), with the cone apex location marked, were fixed in 10% neutral buffered formalin (NBF). Whole corneas were fixed in 2% paraformaldehyde/PBS (pH 7.4). All specimens were paraffin embedded and cut at 6 *μ*m. Sections were collected on Super-Frost Plus slides (Menzel-Glaser, Saarbruckener, Germany) and dried before use.

### 2.2. Immunohistochemistry

Sections were dewaxed and rehydrated through alcohols to water. For antigen retrieval, sections were incubated in 0.01 M citrate buffer (pH 6) at 85°C for 10 minutes, cooled to 40°C, and rinsed in Tris-buffered saline (TBS, pH 7.4) with 0.1% Tween-20 (TBST). Sections were incubated at room temperature (RT) in 5% bovine serum albumin (BSA) in TBST for 30 minutes, followed by incubation overnight at 4°C in HGF or c-Met primary antibodies, or appropriate negative controls (Mouse or Rabbit IgG) (Table 3). After overnight incubation, sections were washed in TBST and incubated in either goat anti-mouse Alexa Fluor 488 or donkey anti-rabbit Alexa Fluor 488.

For co-immunolabelling experiments, a separate series of slides were prepared and HGF visualised with Alexa Fluor 488 and c-Met visualised with Alexa Fluor 594, combined with nuclear Hoechst stain (Table 3).

Immunolabelling was repeated at least twice per specimen for each antibody, and appropriate Ig controls were included for each experiment. All slides were mounted in 20% glycerol/PBS, coverslipped, sealed with nail varnish, and viewed using a Zeiss LSM700 scanning laser confocal microscope and image software (Zen 2011, Carl Zeiss MicroImaging GHBH, Jena, Germany). Where more than one colour was to be detected, multichannel excitation bleed-through was minimized using fluorochromes with separated peak excitations. Emission bleed-through was minimized by multitracking, where signal crosstalk between neighbouring channels was corrected by performing a sequential image capture routine.

### 2.3. Semiquantitative Analysis for HGF and c-Met

Semiquantitative grading of single-immunolabelled sections was used to assess the intensity and distribution of HGF and c-Met immunoreactivity in the corneal epithelium, stroma, and endothelium. Grading for KC buttons was made in the region adjacent to the cone (Adj) and for control corneas in a similar central corneal region. Immunolabelling of the thinned cone area of KC buttons was examined in each specimen but was not graded for comparison with controls because of the obviously altered morphology (only 1-2 epithelial layers present). The grading scale used was based on the intensity of the immunofluorescence (0 = no staining, 0.5 = very weak, 1 = weak, 2 = moderate, and 3 = strong) and the percentage (%) area immunolabelled (0 = 0%, 1 = 1% to 10%, 2 = 11% to 50%, and 3 > 50%) as described previously [19]. A final grade of 0 to 6 (intensity + % area immunolabelled) was then given for each specimen and this data is used to generate frequency histograms for HGF and c-Met immunostaining in KC and control specimens, respectively.

## 3. Results and Discussion

**Figure 1:**
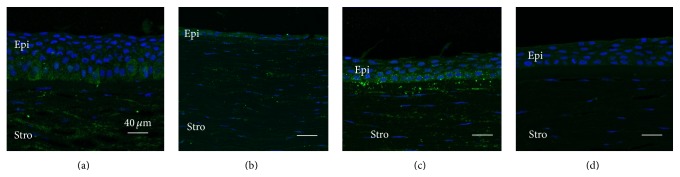
Representative images showing increased HGF expression in basal epithelial cells adjacent to the cone region in KC (a), compared to the relatively more uniform expression seen in all layers of control cornea epithelium at a similar location (c). Strong HGF stromal staining (keratocyte and extracellular matrix) is also observed in KC and control corneas (a to c). No immunostaining is apparent in the Ig negative control (d).* (a) Region adjacent to the cone in KC cornea, (b) cone region in KC cornea, (c) central region of control cornea, and (d) Ig negative control cornea. Epi: epithelium, Stro: stromal. Green: HGF; blue: nuclei*.

**Figure 2:**
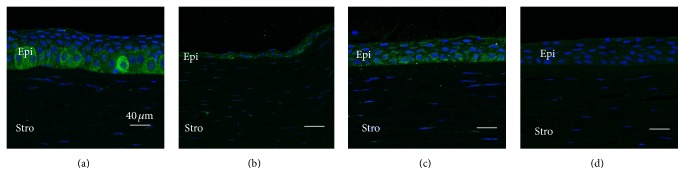
Representative images showing increased c-Met expression in basal epithelial cells adjacent to the cone region in KC (a), compared to the relatively more uniform expression seen in all layers of control cornea epithelium at a similar location (c). Weak c-Met staining is detected in the stroma in both KC and control corneas (a to c). No immunostaining is apparent in the Ig negative control (d).* (a) Region adjacent to the cone in KC cornea, (b) cone region in KC cornea, (c) central region of control cornea, and (d) Ig negative control. Epi: epithelium; Stro: stromal. Green: HGF; blue: nuclei*.

All KC corneas showed a thickened epithelium adjacent to the more central cone region (Figures 1(a) and 2(a)), compared to similar regions in the control corneas (Figures 1(c) and 2(c)), as we previously reported [19]. Only one to two layers of flattened epithelium were observed within the cone region (Figures 1(b) and 2(b)). Weak and more evenly distributed cytoplasmic immunostaining for HGF and c-Met was detected in the epithelium of control corneas (Figures 1(c) and 2(c)). In KC corneas, moderate-to-strong cytoplasmic immunostaining was seen within the basal and wing cell epithelial layers adjacent to the cone region (green fluorescence, Figures 1(a) and 2(a)). Only weak-to-moderate immunostaining of HGF and c-Met was detected in the KC cone region (Figures 1(b) and 2(b)).

**Figure 3:**
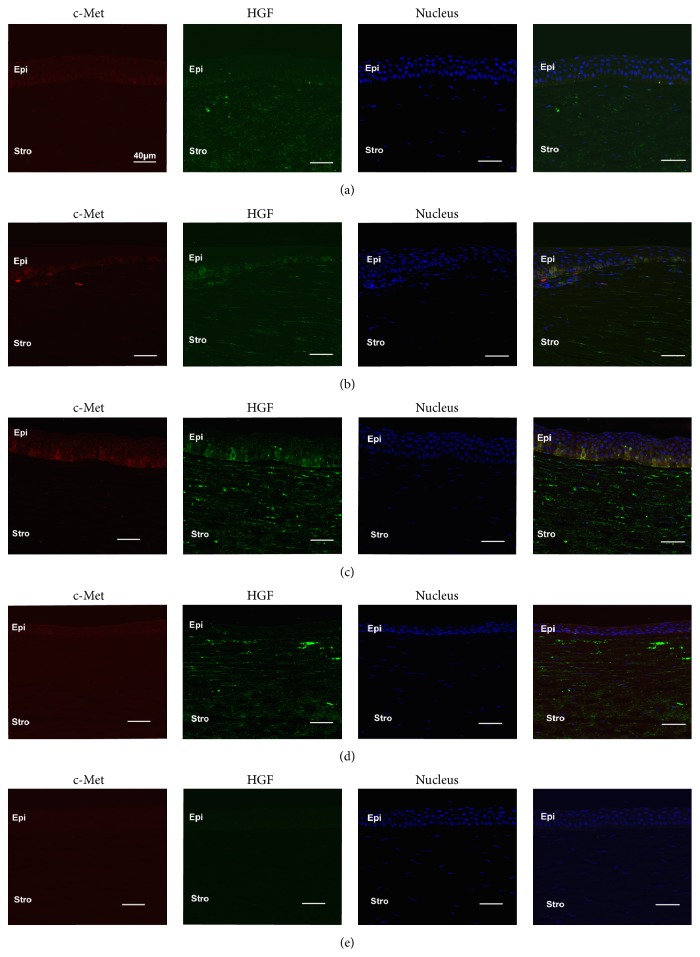
Representative images of central region of corneas with weak, uniform HGF (green), and c-Met (red) expression in all corneal layers (a). Patchy staining of HGF and c-Met is colocalised in the basal epithelium of both limbal regions (b) and adjacent to cone region of KC (c), with KC showing stronger staining than the limbal region. Strong HGF expression is seen within the stroma of both KC (c and d) and control corneas (a and b).* (a) Central region of control cornea, (b) limbal region of control cornea, (c) region adjacent to the cone in KC cornea, (d) cone region in KC cornea, and (e) Ms and Rb Ig control*.

Co-immunolabelling for HGF and c-Met showed that c-Met (red) was primarily localised in the epithelium of both control and KC corneas (Figure 3). HGF (green) was primarily localised within the stroma (both keratocytes and extracellular matrix) of control and KC corneas (Figure 3). Weak and relatively uniform immunostaining was observed in similar regions from control corneas for HGF and c-Met (Figure 3(a)). However, for KC corneas, increased HGF staining (green) was detected in the basal epithelium adjacent to the cone region and colocalised with increased c-Met staining (red) (Figure 3(d)). Limbal areas of control corneas showed slightly stronger stromal immunostaining of HGF compared to the central region of control corneas (Figure 3(b)).

**Figure 4:**
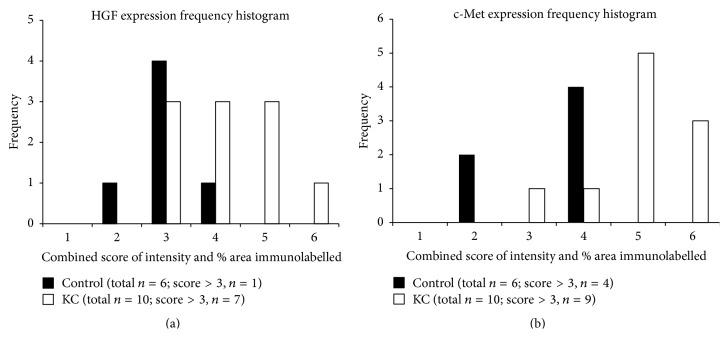
Frequency histograms for HGF (a) and c-Met (b) expression in KC and control corneas (combined score of staining intensity and % area coverage; range = 0 to 6). Note the right skewed distribution of HGF and c-Met immunostaining for KC corneas compared to controls. HGF staining had a combined score >3 in 7/10 (70%) KC corneas, compared to 1/6 (16%) control corneas. c-Met staining with a combined score >3 was seen in 9/10 (90%) KC corneas compared to 4/6 (66%) control corneas.

Semiquantitative grading of HGF and c-Met immunostaining in KC samples was increased overall compared to the control corneas for similar regions, as indicated by the skewed distribution of the frequency histograms (Figures 4(a) and 4(b)). Overall, a greater proportion of KC corneas showed >grade 3 immunostaining for both HGF and c-met (KC: 70% and 90%, resp.,* versus* control: 16% and 66% resp.) (Figures 4(a) and 4(b)).

Despite the importance of the HGF/c-Met pathway in regulating a number of key cellular activities, little is known about its potential role in normal or KC human corneas. As a first step to understand the possible role(s) of this pathway, we examined patterns of HGF and c-Met protein expression in both control and KC corneas. Higher levels of HGF and c-Met immunostaining were detected in the basal epithelium adjacent to the KC cone, compared to the weaker and more uniform epithelial staining pattern seen in control corneas.

In control corneas, we found HGF expression was more intense in the stroma compared to the epithelium. This is consistent with previous studies showing low level* HGF* mRNA expression in the human corneal epithelium compared to keratocytes and endothelium [16, 20]. The surface of normal rabbit corneal epithelium was reported to show only low level HGF protein [21]. Our immunostaining experiments detected stronger c-Met (HGF receptor) staining in epithelium compared to keratocytes in control corneas. This is consistent with* c-Met* mRNA findings, which showed that human corneal epithelium, keratocytes, and endothelium all expressed* c-Met*, but with highest mRNA expression in epithelium [16, 20, 21]. These observations suggest that secreted keratocyte HGF may preferentially bind to the epithelium that expresses higher levels of c-Met (HGF receptor), compared to keratocytes that express low levels of c-Met, thus potentially regulating key epithelial cellular activities such as cell proliferation [22].

HGF is reported to be involved in two major processes, cell proliferation and migration, and inflammatory-related signalling cascades. HGF is a potent enhancer for corneal epithelial cell proliferation [16] and migration [23]* in vivo*, and increased* HGF* and* c-Met* mRNA expressions have been detected during corneal wound healing [16]. Increased HGF protein expression has been detected in the whole corneal epithelium, keratocytes, and tears of wounded compared to unwounded rabbit corneas [21].* HGF* mRNA expression was also upregulated in the lacrimal gland when studying injured compared to unwounded rabbit corneas [17]. No difference in c-Met immunostaining was detected between wounded and unwounded rabbit corneas [21]. In secondary injured KC and control corneas, HGF expression in the stromal cells was reduced [24]. This was suggested to be most likely due to normal tissue repair pathways already being damaged or compromised, implying involvement of HGF in tissue regeneration [24]. These studies also suggested that lacrimal gland and keratocyte-derived HGF are closely associated with corneal tissue repair [17, 24]. In KC corneal epithelium, we observed increased HGF in the region adjacent to the cone, as well as increased c-Met; however it remains to be determined whether the increased expressions of HGF and c-Met observed are directly involved in KC pathogenesis or are secondary responses.

Poorly regulated and overexpressed HGF may be detrimental to tissues, related to the involvement of HGF in inflammation. For example, delayed formation of the epithelium layer and increased formation of stromal myofibroblasts have been detected during rabbit corneal wound healing, for corneas treated with recombinant HGF [18]. Applying recombinant HGF to corneas of mice with* Pseudomonas aeruginosa* keratitis also worsened the disease progression, with a significantly higher grade of corneal opacity and thinning compared to PBS-treated corneas [25].* In vitro* treatment of corneal stromal keratocytes with proinflammatory interleukin- (IL-) 1*β* increased* HGF* mRNA and protein production [26]. Elevated corneal HGF expression also enhanced proinflammatory cytokines and decreased anti-inflammatory cytokines in* Pseudomonas aeruginosa* keratitis in mice, most likely under the control of inflammatory cytokines and cell growth kinases [25].

KC has traditionally been described as a noninflammatory degenerative condition. However, emerging evidence clearly indicates that inflammation-related processes within the epithelium and stroma are involved in the pathogenesis of KC. Significant increases in proinflammatory molecules such as IL-6, IL-4, IL-5, IL-8, and IL-12 [27–29], MMP-1, MMP-3, MMP-7, and MMP-13 [28], and chemokine C-C motif ligand 5 (CCL5) [29] and significantly decreased levels of lactoferrin, serum albumin, and secreted IgA (sIgA) have been reported in KC compared to control tears [30]. Keratocytes in KC are reported to express more IL-1*α* receptors than controls [31], and one group has proposed that keratocyte apoptosis observed in KC may be induced by the binding of IL-1 receptors secondary to increased levels of IL-1 associated with epithelial trauma, for example, due to eye-rubbing [32]. Most recently, increased mRNA expression of* MMP-9* and its inducer proteins IL-6 and TNF-*α* were detected in KC corneal epithelium in an Indian cohort (>100 patients) compared to healthy controls (*n* = 20), and tear protein levels of MMP-9 and IL-6 were also increased in KC [33]. Treatment with topical cyclosporine A (an immunosuppressant) in a small group of KC patients (*n* = 20) reduced the tear levels of MMP-9 and appeared to halt the progression of KC, consistent with the involvement of inflammation in KC pathogenesis [33].

The link between* HGF* variants and KC susceptibility was first reported by Burdon et al., (2011) and then confirmed in an independent European cohort which replicated the association of SNP rs3735520 and detected a new* HGF* SNP rs2956540 [34]. In addition, an independent study of an Australian population of European descent (*n* = 830) focused on the* HGF* locus and detected a different SNP (rs4954218) significantly associated with KC [35]. The actual function of these* HGF *SNPs is unclear. However, a study of rs3735520 implicated a possible regulatory effect of this SNP for HGF protein expression in serum from KC patients [13]. As the SNPs identified to date are all located in the noncoding region of the gene (rs3735520, rs17501108, and rs1014091 in the upstream of* HGF* and rs2286194 in the intron between exon 8 and exon 9), it is likely that they will affect gene expression through mechanisms such as RNA splicing, transcription factor binding, and miRNA regulation [36]. Our results provide evidence of increased HGF protein in KC epithelium compared to control corneal epithelium; however the role of the reported HGF SNPs in the increased protein expression is unclear and will be further investigated.

## 4. Conclusion

Previous studies showed an independent, repeatable association between certain SNPs and* HGF* in KC [13, 34, 35]. The SNP variations detected were located in the noncoding region of* HGF* consistent with a role for these SNPs in the regulating HGF expression, and increased serum HGF expression associated with the minor SNP allele has been reported [37]. In the current study, we showed increased HGF protein expression within KC corneal epithelium. Taken together previous SNP studies, these observations indicate that the HGF/c-Met pathway may be involved in the pathogenesis of KC. Further studies investigating how the HGF/c-Met pathway may be altered in KC, including the associations between SNPs and protein expression and the role of inflammation requires further investigation. Studies on the downstream signalling pathways regulated by HGF/c-Met, such as MAPK cascades, the PI3K-Akt axis, and the JAK/STAT pathway may help to identify the potential role of this pathway in KC and in normal corneal homeostasis.

## Figures and Tables

**Table 1:** Characteristics of KC patients.

^a^KC	Gender	Age at diagnosis (yrs.)	Age at surgery (yrs.)	Contact lenses (Y or N)	History of allergy and/or atopy (Y or N)	^b^DALK (Y or N)
1	F	24	32	Y	N	N
2	F	23	30	N	Y asthma	Y
3	F	30	37	N	N	Y
4	M	27	32	N	Y atopy and asthma	N
5	M	25	32	N	N	Y
6	F	18	21	N	Y atopy and asthma	N
7	M	24	28	Y	N	N
8	M	24	43	Y	N	N
9	F	20	31	Y	N	N
10	F	32	38	Y	N	Y

^a^Grade 4 KC: severe; VA >6/7.5 with contact lens correction; severe corneal thinning and Munson's sign.

^b^DALK: deep anterior lamellar keratoplasty.

**Table 2:** Characteristics of control corneas.

Control	Gender	Age (yrs.)
1	M	67
2	F	53
3	F	64
4	F	67
5	M	83
6	M	63

**Table 3:** Primary antibodies and negative controls used for immunostaining.

Primary antibody	Company	Final concentration used
Mouse anti-human HGF	Santa Cruz Biotechnology, Inc. (Dallas, Texas, USA)	2 *µ*g/mL
Rabbit anti-human c-Met	Santa Cruz Biotechnology, Inc.	0.4 *µ*g/mL
Mouse anti-human IgG	Zymed Laboratories (Thermo Fisher Scientific, Waltham, Massachusetts, USA)	2 *µ*g/mL
Rabbit anti-human IgG	Zymed Laboratories	0.4 *µ*g/mL
Alexa Fluor 488 goat anti-mouse IgG	Molecular Probes (Life Technologies, Carlsbad, California, USA)	2 *µ*g/mL
Alexa Fluor 488 donkey anti-rabbit IgG	Molecular Probes	2 *µ*g/mL
Alexa Fluor 594 donkey anti-rabbit IgG	Molecular Probes	2 *µ*g/mL
Hoechst	Molecular Probes	1 *µ*g/mL
