# Corrigendum to “Expression of HGF and c-Met Proteins in Human Keratoconus Corneas”

**DOI:** 10.1155/2016/4201505

**Published:** 2016-08-17

**Authors:** Jingjing You, Li Wen, Athena Roufas, Chris Hodge, Gerard Sutton, Michele C. Madigan

**Affiliations:** ^1^Save Sight Institute, Discipline of Clinical Ophthalmology, The University of Sydney, Sydney, NSW 2000, Australia; ^2^Vision Eye Institute, Chatswood, NSW 2067, Australia; ^3^Auckland University, Auckland 1010, New Zealand; ^4^School of Optometry & Vision Science, UNSW, Kensington, NSW 2052, Australia

In the article titled “Expression of HGF and c-Met Proteins in Human Keratoconus Corneas” [1], there was an error in the “Acknowledgments” section, which should be corrected as follows:

The authors thank the Medical School of Sydney University, The Ophthalmic Research Institute of Australia (ORIA), Sydney Eye Hospital Foundation, and the Lions NSW Eye Bank for funding support. MCM is funded by the National Foundation for Medical Research & Innovation (NFMRI).
